# Development of a New Equation for the Prediction of Resting Metabolic Rate in Sri Lankan Adults

**DOI:** 10.1155/2021/4170137

**Published:** 2021-01-20

**Authors:** Pathima Fairoosa, Pulani Lanerolle, Maduka De Lanerolle-Dias, V. Pujitha Wickramasinghe, Indu Waidyatilaka

**Affiliations:** ^1^Department of Biochemistry and Molecular Biology, Faculty of Medicine, University of Colombo, PO Box 271, Kynsey Road, Colombo 8, Sri Lanka; ^2^Department of Paediatrics, Faculty of Medicine, University of Colombo, PO Box 271, Kynsey Road, Colombo 8, Sri Lanka

## Abstract

Resting metabolic rate (RMR) is the key determinant of the energy requirement of an individual. Measurement of RMR by indirect calorimetry is not feasible in field settings and therefore equation-based calculations are used. Since a valid equation is not available for Sri Lankans, it is important to develop a new population-specific equation for field use. The study objective was to develop a new equation for the prediction of RMR in healthy Sri Lankans using a reference method, indirect calorimetry. RMR data were collected from fifty-seven (male 27) adults aged 19 to 60 years. They were randomly assigned to validation (*n* = 28) and cross-validation (*n* = 19) groups using the statistical package *R* (version 3.6.3). Height, weight, and RMR were measured. Multivariable fractional polynomials (MFP) were used to determine explanatory variables and their functional forms for the model. A variable shrinkage method was used to find the best fit predictor coefficients of the equation. The developed equation was cross-validated on an independent group. Weight and sex code (male = 1; female = 0) were identified as reliable independent variables. The new equation developed was RMR (kcal/day) = 284.5 + (13.2 x weight) + (133.0 x sex code). Independent variables of the prediction equation were able to predict 88.5% of the variance. Root mean square error (RMSE) of the prediction equation in validation and cross-validation was 88.11 kcal/day and 79.03 kcal/day, respectively. The equation developed in this study is suitable for predicting RMR in Sri Lankan adults.

## 1. Introduction

The prevalence of obesity is increasing in Sri Lanka [1], which is a major risk factor for diet-related noncommunicable diseases including type 2 diabetes mellitus, cardiovascular diseases, and hypertension [2]. It is due to a positive energy balance where energy intake exceeds energy expenditure [2]. Total energy expenditure (TEE) is the sum of resting metabolic rate (RMR), thermic effect of food, and physical activity energy expenditure [3]. RMR is the amount of energy the body expends at rest and it accounts for approximately 60% to 70% of the TEE [2]. It is used for the estimation of the energy requirement of an individual [2]. Achieving balance between energy intake and expenditure is key to optimum body weight [4]. This requires knowledge of an individual's energy requirements and relies on accurate methods of assessment [5].

RMR can be measured using direct or indirect calorimetry. However, the use of these methods at field level is not feasible due to high cost and the need for trained personnel [5]. Since calorimeters to measure RMR in field settings are limited, it would be prudent to use other methods such as equations to estimate RMR [5]. Prediction equations are developed from RMR data collected using a reference method and independent variables, such as body weight, height, age, and sex [3].

Equations for the prediction of RMR have been developed in different populations during the last century following the early work of Harris-Benedict in 1918. A recent review reporting on 248 prediction equations showed that most of these equations for the prediction of RMR were developed on Western populations [6]. The validity of their usage on other populations has been repeatedly questioned and these equations have been cross-validated in different populations [7, 8]. Prediction equations are accurate in a population which would closely match the original population used to develop the equation [9]. Several Asian studies revealed that most commonly used equations for the prediction of RMR such as Harris-Benedict [10], Schofield [11], FAO/WHO/UNU [12], and Mifflin et al. [13] overestimated the RMR in Asians [7, 8]. Therefore, it is important to develop appropriate equations for different ethnic groups when possible. Unsuitability of existing equations for the prediction of RMR in Asians led to the development of new equations in Asian populations. Soares et al. [14] and Piers and Shetty [15] developed two equations to predict RMR in Indian males and females, respectively, in 1993. To the best of our knowledge, these two are the only equations developed on a South Asian population.

Currently, there is no population-specific equation available for Sri Lankans to predict RMR. It is important to either validate an existing equation on the target population or if the existing equations are not valid, develop a new population-specific equation for the target population. In the present cohort, ten selected equations including Harris-Benedict, Schofield, Mifflin St. Joer, Owen, and WHO and equations developed on Asians and Indians demonstrated significant overestimation in predicting RMR [16]. Unsuitability of the existing equations for the prediction of RMR for Sri Lankan adults highlighted the need for development of a new population-specific equation [16]. This study aimed to develop a new equation for the prediction of RMR in healthy Sri Lankan adults using the reference method, indirect calorimetry.

## 2. Materials and Methods

### 2.1. Study Participants

A total of 57 healthy volunteers (27 males) aged between 19 and 60 years, residing in the Western province, were recruited to this cross-sectional study. Sample size was calculated according to Knofczynski and Mundfrom method for prediction model study [17]. Pregnant or lactating mothers, those who have attempted to lose weight over the past three months, those who were having a major illness or on medication, and those who have had bariatric surgery, were excluded from the study. This study was carried out at the Department of Biochemistry and Molecular Biology, Faculty of Medicine, University of Colombo, Sri Lanka.

### 2.2. Anthropometric Measurements

A single trained investigator did all anthropometric measurements under standard conditions using the International Society for Advancement of Kineanthropometry (ISAK) protocol [18]. Weight was measured under standard conditions to the nearest 0.1 kg using a calibrated electronic weighing scale (Seca 803, Seca GMBH & Co., Kg., Germany). Height was measured under standard conditions to the nearest 0.1 cm using a stadiometer (Seca 225, telescopic height measurement, Seca GMBH & Co., Kg., Germany). Body mass index (BMI) was calculated as weight/height^2^ (kg/m^2^).

### 2.3. RMR Measurement

RMR was measured using an open circuit desktop indirect calorimeter (Cosmed Fitmate GS^®^, Italy). Subjects were instructed to fast for a minimum of 10 hours and also to abstain from alcohol, caffeine, nicotine, and vigorous exercises for 12 hours before the test. Female participants were advised not to schedule their sessions during menstruation days to avoid any effect of menstruation on RMR. A single investigator performed the calorimetric measurements according to standard protocol [19]. The participants were asked to rest for 20 minutes before the test in an air conditioned room at 25°C at supine position. RMR measurements were performed between 8 and 10 a.m. while participants were at rest in the supine position. The test was done in a thermoneutral room at a temperature of 25°C, ensuring that each individual was physically comfortable and properly positioned for measurements. Data obtained within the first five minutes were discarded and the next 20 minutes steady-state data were recorded according to the standard protocol. RMR measurement was repeated seven days later under similar condition for each participant and mean RMR was used for the analysis.

The Cosmed Fitmate GS^®^ system contains a transparent plastic canopy hood that covers the subject's head and has a continuous stream of room air. The canopy hood was attached to a flow meter (bidirectional turbine). The stream of ambient air dilutes the participants' expired gas, which is directed to the oxygen analyzer to determine the VO_2_ and RMR. Cosmed Fitmate GS^®^ system uses 0.85 as the respiratory quotient to calculate RMR using the modified Weir equation, [EE (kcal/day) = ([VO_2_ × 3.941] + [0.85 × VO_2_ × 1.11]) × 1440] [20]. The gas analyzer and the flow meter were calibrated before each session by a 3-litre calibration syringe (Fitmate, Rome, Italy).

### 2.4. Statistical Analysis

Data of all fifty-seven participants were included in the final analysis. All statistical analysis was carried out using the statistical package *R* version 3.6.3. A data-splitting algorithm was used to randomly separate the data into validation and cross-validation datasets in a ratio of 2 : 1. Parameters were compared between validation (*n* = 38) and cross-validation (*n* = 19) groups using independent samples *t*-test. The significance was set at *p* < 0.05. The validation dataset was used to construct our prognostic model and to estimate the variable coefficients; the cross-validation data were used to validate the model.

Normality and multicollinearity of the variables were evaluated. Association between dependent and independent variables were assessed by plotting scatter plots with regression line. Binary dummy variables (male = 1 and female = 0) were used to code sex [21]. Multivariable fractional polynomials (MFP) were fitted for continuous variables (height, weight, BMI, and age). Fractional polynomials are a method for fitting more flexible polynomials than the usual simple polynomials and involve selection from a set of polynomial functions. Multivariable fractional polynomial fitting is based on a closed-test procedure that maintains an overall type 1 error (alpha level) of 0.05 for tests. These variables were fitted using closed test to examine whether they should be included or excluded using *α*1, and whether fractional transformation should be performed using *α*2 [22]. Explanatory predictor variables and functional forms were determined using “mfp” package in *R* version 3.6.3 in the validation group.

Variable shrinkage was used to estimate reliable predictor coefficients of the developed equation to reduce the risk of overfitting. Different penalties were applied to find the equation with the highest prediction accuracy. Predictor coefficients derived from least square regression were brought closer to zero by multiplying by a constant in ridge regression. This method keeps all predictors in the final model. Coefficients were brought to zero by adding or subtracting a constant in least angle selection and shrinkage operator (LASSO) method. This method ensures sparsity of the results by shrinking some coefficients to zero. Elastic net is a hybrid of ridge regression and LASSO by adjusting the values of hyperparameter *α* [23].

The best prediction model among different penalty terms was selected by the measure of goodness-of-fit statistic. The best prediction equation should have a high *R*^2^ with minimum root mean square error (RMSE). The best model was cross-validated in an independent sample [21, 24]. Bland–Altman method [25] was used to evaluate mean difference between two measurement methods (the bias) and 95% limits of agreement as the mean difference (±1.96 SD).

### 2.5. Ethics

The Ethics Review Committee of the Faculty of Medicine, University of Colombo, approved the study protocol (EC-18-68), and all procedures followed were in accordance with the ethics standards of this committee. Informed written consent was obtained from all participants.

## 3. Results

The general characteristics of the participants in the validation group and cross-validation group are given in Table 1. The study population included 57 adults, of which 47% were males. The mean RMR measured in males and females were 1291.3 ± 165.6 kcal/day and 1060.2 ± 122.9 kcal/day, respectively. There was no significant difference in age, anthropometry, and RMR data between validation and cross-validation.

**Figure 1:**
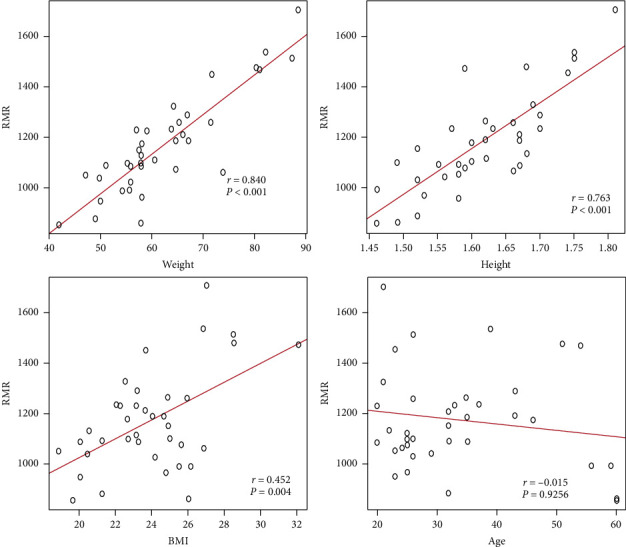
Association between RMR and continues independent variables in validation group.

All the continuous variables were normally distributed. RMR by indirect calorimetry (reference method) was significantly correlated with weight (*r* = 0.840, *p* < 0.001), height (*r* = 0.763, *p* < 0.001), and BMI (*r* = 0.452, *p*=0.004) of the validation group. Age (*r* = -0.015, *p*=0.9256) did not show a significant correlation with RMR as shown in Figure 1. RMR was the dependent variable and weight, height, BMI, age, and sex were possible independent predictors.

MFP results are shown in Tables 2 and 3. Among the variables considered, weight, height, BMI, and age were the continuous variables. FP (FP2, degrees of freedom (df) = 2) functions were applied for these continuous variables with the intention of including variables that have nonlinear associations with RMR yet a reliable and significant predictor of RMR. MFP test resulted in a generalized linear model that includes weight and gender. Weight and gender were reliable predictors of RMR. Height, BMI, and age were not included in the prediction model and were not predictors of RMR in this population.

**Figure 2:**
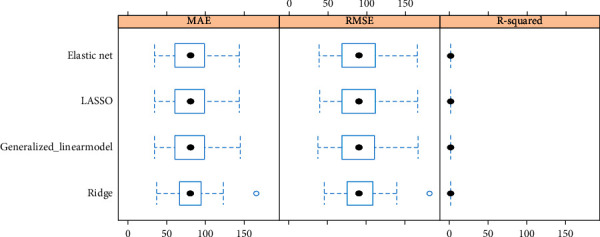
Comparison between the methods (generalized linear, LASSO, elastic net and ridge).

Variable shrinkage (ridge, LASSO, and elastic net regression) with 10-fold repeated cross-validation method was used to estimate reliable predictor coefficients for the final model. Best model fit with least RMSE and highest *R*^2^ was obtained by elastic net method that linearly combines the L^1^ and L^2^ penalties of the LASSO and ridge methods. Comparison between the methods is shown in Table 4 and Figure 2.

Prediction coefficients were obtained for best fit elastic net regression model where alpha was 0.1 and lambda was 1. The final model was RMR (kcal/day) = 284.5 + (13.2 x weight) + (133.0 x sex code). *R*^2^ and RMSE obtained for the final model were 0.88, RMSE = 88.11 kcal/day, respectively. Final model was cross-validated in an independent cross-validation sample (*n* = 19). RMSE obtained for the cross-validation was 79.03. There was no significant difference between RMR by indirect calorimetry and RMR by the preliminary equation (1162.3 kcal/day and 1146.3 kcal/day, *p*=0.767). RMSE obtained from the cross-validation group and validation groups were comparable.

**Figure 3:**
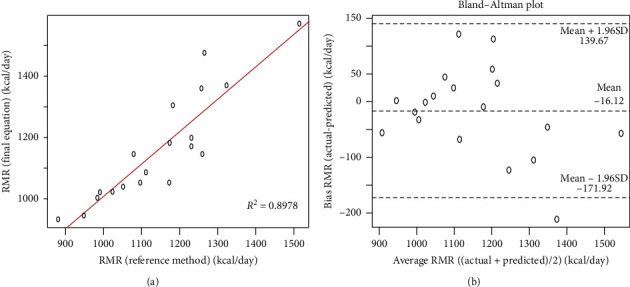
Association between RMR by indirect calorimetry (reference method) and RMR by prediction equation. (a) Regression line for RMR (*R*^2^ = 0.89, *p* < 0.001). (b) Bland–Altman plot difference for RMR (*r* = 0.35, *p*=0.1417).

RMR predicted by the prediction equation was plotted against RMR assessed by the reference method IC (Figure 3(a)). The Bland–Altman plot between the equation and RMR by the reference method for plot difference is shown in Figure 3(b). The limit of agreement was +139.67 to −171.92 and the equation resulted in a small mean negative bias of 16.12 ± 79.48 kcal/day. Association between RMR bias and mean RMR was not significant (*r* = 0.35, *p*=0.1417).

## 4. Discussion

The new equation developed and validated for the prediction of RMR in Sri Lankan adults fills a significant void in accurate but simple prediction methods for RMR. The independent variables included in the prediction equation showed a higher degree of association with the dependent variable than the variables that were not included. RMR measured by indirect calorimetry and RMR predicted by the equation correlated well. When the equation was cross-validated in an independent sample, it had a small bias and narrow limit of agreement, indicating very good performance.

Cross-validation of existing equations, Harris-Benedict, Schofield, WHO weight, WHO height and weight, Owen, Mifflin St. Jeor, Henry, Liu, Ganpule and Indian equations developed by Soares et al. [14] and Piers and Shetty [15], were previously performed in the present cohort [16]. All the equations significantly overestimated RMR in Sri Lankan adults and the minimum bias reported (−170 ± 102 kcal/day) was reported for the Ganpule equation. The equation developed in this study is more robust and had smaller bias (−16.12 ± 79.48 kcal/day) than existing equations in the present cohort.

MFP approach was adopted in the present study to adjust the continuous variables (weight, height, BMI, and age), allowing for nonlinear functional forms. Weight and gender were the independent variables selected by MFP as the potential variables to predict RMR in this study. Allowing for polynomial functional forms in the present study, height, BMI, and age were not included in the model suggesting that these variables and their polynomials were not significant predictor variables. We used a variable shrinkage method to avoid the effect of overfitting and multicolinearity in the model. Predictor coefficients were tuned by ridge, LASSO, and elastic net regression in order to improve the predictability of the generalized linear model resulting from MFP. Variable shrinkage methods reduce the sampling variation and improve the generalizability of the model. Elastic net regression resulted in the best fit model. Predictor coefficients were estimates for this best fit model (prediction equation). Bias RMR and average RMR were not significantly correlated with each other suggesting that the equation does not result in higher bias for higher RMR and lower bias for lower RMR.

The RMR value of 1169 ± 184 kcal/day measured using indirect calorimetry in our study was comparable with values reported for Asians and South Asians. Piers and Shetty [15] reported 1122 ± 143 kcal/day (*n* = 60) RMR for an Indian female, and Rao et al. [7] reported 1384 ± 285 kcal/day (*n* = 21) and 1094 ± 238 kcal/day (*n* = 22) for young Chinese male and female, respectively [7]. It is well known that RMR in Asians is lower than that of the Western population [7, 8, 26]. The lower RMR in Asians could be explained by ethnic variations in body composition. Asians are prone to have more adipose tissue compared to the Western population for a given body weight, thus having less muscle mass for a given weight. [27, 28]. RMR is related to muscle mass which is the metabolically active tissue.

In this study, weight was the strongest predictor of RMR and had the highest correlation (*r* = 0.840, *p* < 0.001). This finding is in agreement with almost all the previously published studies on RMR, where body weight accounts for the highest variation in RMR [9, 26, 29]. RMR is affected by several factors other than body weight. These factors are sex, height, BMI, age, and body composition, particularly metabolically active tissue [27]. Sex, height, and BMI were positively correlated with RMR measured by indirect calorimetry in our study. However, in the development of the prediction equation, while height and BMI were noncontributing variables, weight and sex were the strongest predictors of RMR included as independent variables. RMR values in males are generally higher than in females and again are likely due to the differences observed in the amount of metabolically active tissue such as muscle [2]. In our study, we observed that mean RMR in males was 1291 ± 166 kcal/day and mean RMR in females was 1060 ± 123 kcal/day. Age was not significantly correlated with measured RMR (*r* = −0.015, *p*=0.9256) in our study though previous studies have reported its negative correlation with RMR [2]. This can be explained by the fact that our study had a small number of participants for a given age range. This number may not be adequate enough to capture a significant negative relationship between age and RMR.

Most regression equations are based on easily measurable anthropometric parameters (body weight, height, and age) while some are based on body composition parameters (total body water (TBW), fat mass (FM), and fat-free mass (FFM) [30]. FFM has been a recent focus of metabolic research due to its potential role in the development of noncommunicable diseases (NCD) such as diabetes mellitus [2]. If FFM has a protective role in the development of NCDs, with potential likely mechanisms being through its effect on metabolic rate, the study of a FFM-RMR relationship becomes important. FFM can be found in some RMR prediction models [2, 9]. A recent study compared weight and FFM in a different model and concluded that including FFM instead of body weight had a slight improvement (*R*^2^ = 0.702 vs *R*^2^ = 0.706) in prediction accuracy [9]. However, accurate body composition assessment requires equipment and training and including them in RMR prediction models, especially in a resource-poor setting, may reduce its feasibility of use in field settings [30].

The equation developed in our study was a combined sex code, weight-based equation, which can easily be used at field level. The equation developed by Almajwa and Abulmeat [9] included FFM, TBW, FM, and age and was developed in a larger population and the mean bias reported was comparable with our equation. Our equation has the simplicity of only having a weight measurement and sex as predictors while preserving accuracy.

Physical activity and sedentary behaviour could have an impact on RMR [30]. In our study, any short-term influence of physical activity was minimized through ensuring that participants refrained from vigorous exercise immediately before the test day. The strength of this study is that the requirements of RMR measurement (postabsorptive state, absence of muscular activity, and thermoneutral environment) were strictly met. Further possible confounding factors such as menstruation, pregnancy and lactation, and known major illness were accounted for through exclusion criteria.

The major limitation of this study is the use of Fitmate GS^®^ desktop indirect calorimeter as the reference method. Fitmate GS^®^ system does not contain a CO_2_ sensor and it calculates CO_2_ production from O_2_ production by assuming 0.85 as the respiratory quotient (RQ). However, scientific evidence indicates that in a steady state, RQ is in the range of 0.84 ± 0.04 [3]. Therefore, the assumption (RQ = 0.85) may introduce little error in estimating RMR. Other limitations of this study were the small sample size and that participants were predominantly from the Western province, which may reduce the generalizability of the results to the whole country without further validation. Future studies should be designed to cross-validate the new equation in different populations to confirm the accuracy and applicability of this equation.

## 5. Conclusion

A new population-specific equation for the prediction of RMR was developed for Sri Lankan adults. The new equation performed well in Sri Lankan adults and showed good agreement with measured RMR. Therefore, this equation is suitable for the prediction of RMR in Sri Lankan adults. The newly developed equation is simple; its variables, combined sex code and weight, make it easy to use at field level.

## Figures and Tables

**Table 1:** General characteristics of validation and cross-validation group by sex.

N	Validation group	Cross-validation group	*p* value
	38 (mean ± SD)	19 (mean ± SD)	
Age (years)	34.6 ± 13.2	35.4 ± 12.9	0.8255^1^
Weight (kg)	63.0 ± 10.4	62.6 ± 10.4	0.6272^1^
Height (cm)	162.4 ± 8.3	161.5 ± 9.6	0.7176^1^
^2^BMI (kg/m^2^)	23.8 ± 3.0	23.5 ± 2.5	0.6886^1^
^3^RMR (kcal/day)	1181.3 ± 199.7	1146.2 ± 152.3	0.4653^1^

^1^Differences between validation and cross-validation groups were analysed using independent sample *t*-test. ^2^Body mass index. ^3^Resting metabolic rate.

**Table 2:** Multivariable fractional polynomials (MFP) output.

Variable	Deviance	Power (s)
*Cycle 1*		
Weight	288296.9	—
	250396.2	1
	221165.6	−1
	212826	2, 1
Gender 1	280378.9	—
	250396.2	1
BMI	268815.4	—
	250396.2	1
	220161.5	−0.5
	206013.9	3, 3
Height	277688.7	—
	268815.4	1
	266702.9	3
	261586.7	3, 3
Age	294960.8	—
	277688.7	1
	276986.6	−1
	276324.9	−2, 3

*Cycle 2*		
Weight	884932	—
	294960.8	1
	257869.8	3
	255975.6	3, 3
Gender 1	433506.4	—
	294960.8	1
BMI	294960.8	—
	262089.8	1
	243042.8	−2
	231763.8	3, 3
Height	294960.8	—
	270268.1	1
	267832	3
	263642.4	3, 3

**Table 3:** Fractional polynomials

Fractional polynomials	df. initial	Select	Alpha	df. final	Power1	Power2
Weight	4	0.05	0.05	1	1	—
Gender	4	1	0.05	1	1	—
BMI	4	0.05	0.05	0	—	—
Height	4	0.05	0.05	0	—	—
Age	4	0.05	0.05	0	—	—

**Table 4:** Comparison between the models (generalized linear, LASSO, elastic net, and ridge).

Method	Mean absolute error (MAE)	RMSE	*R* ^2^
Elastic net	81.74	91.83	0.885
LASSO	81.66	91.84	0.885
Generalized linear model	81.75	91.85	0.885
Ridge	81.25	92.34	0.879

## Data Availability

Data used to support the findings of this study are available from the corresponding author upon request.
